# Supplementary material for: Bone metastasis and skeletal-related events in patients with solid cancer: A Korean nationwide health insurance database study
Source: PLoS One. 2020 Jul 17;15(7):e0234927. doi: 10.1371/journal.pone.0234927 (PMC7367479; doi:10.1371/journal.pone.0234927)
Supplement: S1 Table — (DOCX) [file pone.0234927.s001.docx]

| **S1 Table. Cumulative risk of SREs (%)** | | | |
| --- | --- | --- | --- |
| Cancer type | 1 year | 3 year | 5 year |
| Stomach | 32.6 | 43.7 | 61.1 |
| Colorectal | 36.1 | 55.0 | 65.1 |
| Liver | 44.5 | 52.0 | 65.9 |
| Lung | 47.8 | 53.7 | 66.4 |
| Breast | 32.1 | 46.8 | 59.5 |
| Prostate | 29.9 | 51.5 | 67.0 |
| other GU | 30.4 | 44.9 | 59.9 |
