# Supplementary material for: Bone metastasis and skeletal-related events in patients with solid cancer: A Korean nationwide health insurance database study
Source: PLoS One. 2020 Jul 17;15(7):e0234927. doi: 10.1371/journal.pone.0234927 (PMC7367479; doi:10.1371/journal.pone.0234927)
Supplement: Attachment — Submitted filename: Response to Reviewers.docx [file pone.0234927.s002.docx]

Reviewer #2:

1. Which ICD-code of bone surgery SRE in this study?

Thank you for comment. I added procedure codes for bone surgery in study population.

2. The literature showed that once a patient experiences an SRE, the risk of subsequent SRE is increase. I wonder if this study had the data on the cumulative number of SREs in each patient?

Thank you for your comment. I agree with your opinion, however we focused only on the first event of each SRE in this paper. We do not have information about cumulative number of SREs.

3. Pathologic fracture is strongly associated with an increased risk of death. How about the differences of overall survival between BM patients with fracture SRE and those without fracture SRE, and those without SRE.

Thank you for your comment.

We tried to compare the overall survival according to each SRE, but the proportion of death in each group was small and we could not find any meaningful results.

4. In the discussion part, please correct "1,845 bone metastasis patients" into "1,849".

Thank you for your comment. I corrected it.

Reviewer #3:

The definition of SRE (Skeletal-related events) about radiation is slightly different for each paper. (severe bone pain requiring radiation vs. just radiation to bone)
In some cases, radiation therapy is administered for asymptomatic bone metastases in patients to reduce the risk of skeletal-related events. Therefore, it must be noted and the authors should concern and describe about this.

Thank you for your comment.

We added your comment for the weaknesses of the discussion. Two reasons for radiation cannot be distinguished from claims data.

“In the definition of radiation therapy, we could not distinguish the reason for radiation therapy because we used the claim data. The radiation therapy is usually performed when bone pain is present, but in some cases, it is also used for asymptomatic patients to reduce the risk of SRE. “

Reviewer #4:

1. In material and methods section. I can’t easily understand the following sentences. Please explain. “We excluded paticipants who were aged less than 20 years~ at least 2 years to assess for bone metastases and SREs” What does washout mean?

Our database is from 2002. In the first year, usually we use wash out period for cancer diagnosis because previous prevalent cases may confound incidence. We revised the sentence;

“We used a 1-year washout period to exclude cancer patients who had been diagnosed in the past.”

2. Please add Kaplan-Meier survival curves for OS, and survival after bone metastasis if possible. (You can add just 2 figures, one for OS including all cancer types, and the other for survival after bone metastasis)

Thank you for your comment.

We added two figures.

Reviewer #5:

1. This study focused on 7 major cancer types (stomach, colorectal, liver, lung, breast, prostate, and other genitourinary cancers). The reference about Korea cancer incidence should be added.

We inserted reference about cancer statistics in Korea.

2. Why did authors use the only C795 for bone metastases? The reason should be documented in discussion.

C795 is ICD-10 code for bone metastasis. We added an explanation for the code.

3. Authors defined pathologic fracture, spinal cord compression, bone radiation, and bone surgery as SREs. Why did authors exclude hypercalcemia from SREs?

Thank you for your comment. Unfortunately, in health insurance claim data, it was impossible to retrieve the value of hypercalcemia. In terms of definition of SRE, some papers included hypercalcemia, but some papers did not include hypercalcemia depending on authors.

And what was the procedure cord for bone surgery?

We added procedure codes for bone surgery in study population.

How did authors handle overlapping data from the different cords?

We count 1^st^ SRE event as index event for individual patient.

I don’t understand this sentence “In addition, patients diagnosed in 2002 were excluded for washout and were followed up for at least 2 years to assess for bone metastases and SREs”.

Our database is from 2002. In the first year, usually we use wash out period for cancer diagnosis because previous prevalent cases may confound incidence. We revised the sentence;

“We used a 1-year washout period to exclude cancer patients who had been diagnosed in the past.”

4. In result, authors commented that bone mets increased with age and there was no difference in region or household. Statistical p value should be added. If authors did not use statistical analysis, the content should be corrected.

Thank you for your critical comments. As your advice, we added statistical p value in table 2 and corrected my manuscript.

“Bone metastases showed an increasing trend in rural areas compared to that in urban areas. Household income had no impact on the occurrence of bone metastasis.”

5. Discussion is ambiguous and extensive. This cloud the issue. Please clarify authors’ suggestion and present specified contents.

Thank you. We revised the discussion part.

6. The manuscript is not easy to read and the English used really should be checked for grammar and spelling.

We requested English proofreading once again and submit it with completion. Thank you.
